# The effect of cognitive behavioral counseling on anxiety and worry level of women with intermediate risk during first trimester screening for down syndrome: a randomized controlled trial: a randomized controlled trial

**DOI:** 10.1186/s12884-023-05857-2

**Published:** 2023-09-01

**Authors:** Roghieh Kharaghani, Farhaneh Vaezi, Mohsen Dadashi, Leila Rastegari, Azam Maleki

**Affiliations:** 1 Department of Midwifery, School of Nursing and Midwifery, Zanjan University of Medical Sciences, Zanjan, Iran; 2 Department of Psychology, Zanjan University of Medical Sciences, Zanjan, Iran; 3 Social Determinants of Health Research Center, Zanjan University of Medical Sciences, Zanjan, Iran

**Keywords:** Cognitive- behavior consulting, Anxiety, Worry, Pregnant, Fetal chromosomal abnormalities

## Abstract

**Background:**

Anxiety related to prenatal screening programs negatively affects maternal and child health.

**Objective:**

The study aimed to determine the effect of Cognitive Behavioral Counseling on the anxiety and worry levels of women with intermediate risk during first-trimester screening for Down Syndrome.

**Methods:**

The study was a randomized controlled trial conducted on 52 pregnant women with intermediate risk (1: 51 − 1:1500) during first-trimester screening for Down Syndrome and without additional structural anomalies that referred to three cities of Zanjan province in 2021. The eligible women were randomly assigned to intervention and control groups, with a block size of four. The intervention group received CBC in four sessions of 120 min two times a week by phone. Data were collected using Vandenberg Anxiety Questionnaire, and Cambridge Worry Questionnaire in three phases baseline, after the intervention, and 6 weeks follow-ups. Data were analyzed using independent t-test, chi-square, and repeated measures ANOVA at a 95% confidence level. (P < 0.05).

**Results:**

In the counselling group, the mean (SD) of a total score of anxiety before the intervention was 67.11 (20.68) which decreased to 32.50 (13.58) in six weeks after the intervention. Furthermore, the mean (SD) of a total score of worry before the intervention was 56.19 (16.76) which decreased to 32.96 (8.89) six weeks after the intervention. Based on the repeated measures ANOVA test, the mean total score of anxiety and worry were statistically significant 6 weeks after the intervention compared with the control group(p < 0.001).

**Conclusion:**

Based on the study results, CBC can reduce the anxiety and worry levels of women with intermediate risk during first trimester screening for Down Syndrome.

**Trial registration:**

The study was registered at the Iranian Registry of Clinical Trials website under the code IRCT20160608028352N8, (https://en.irct.ir/trial/49998). The first trial registration date was (29/08/2020).

**Supplementary Information:**

The online version contains supplementary material available at 10.1186/s12884-023-05857-2.

## Introduction

Congenital anomalies are defined as abnormalities in the structure or function of the body that exists at birth and have a prenatal origin. According to the Global Burden of Disease study by the World Health Organization (WHO), 17 to 42% of neonatal mortality occurs due to birth defects [1]. Worldwide, it is estimated that more than 5 million babies are born with birth defects each year. According to the WHO 2020 report, 295,000 deaths occur each year in the first 28 days after birth due to congenital anomalies [2]. The overall prevalence of congenital anomalies in Iran is estimated to be 2.6% [3], and in Zanjan 0.6 to 0.7% [4]. Therapeutic abortion refers to a deliberate ending of a pregnancy that is carried out or approved by a doctor to preserve the health and life of the mother. In Iran, it is lawful to terminate a pregnancy if three gynaecologists agree that the continuation of the pregnancy would pose a danger to the mother’s health during the pregnancy or after giving birth, or if there is a significant fetal abnormality. However, the existing legal and religious frameworks in Iran restrict the granting of permission for pregnancy termination to be carried out only before the 19th gestational weeks according to the last menstrual period (LMP) [5].

Although therapeutic abortion is legally permitted in Iran, its acceptance and availability in practice may still be influenced by societal and cultural factors. One of the main factors is the strong influence of religious beliefs, where some individuals may view the termination of pregnancy as morally wrong. This can lead to stigmatization and social pressure against women who choose to undergo the procedure, which may make it difficult for them to access safe and legal abortion services [6].

Nowadays, advances in clinical trials and technological improvements have made it possible to diagnose fetal anomalies during pregnancy and before birth [7]. A prenatal screening program identifies pregnant women at risk of having a baby with major chromosomal anomalies. This, in addition to the many benefits, provides grounds for anxiety and worry for pregnant mothers. According to some studies, fetal health is one of the factors that cause the most concern in pregnant mothers [8].

Worry is a part of everyday human experiences and an important component of anxiety that is formed due to the prediction of unpleasant events in the future. Anxiety is the worry in advance about future dangers, along with the physical symptoms of stress, that result from getting ready for events that are considered dangerous [9]. According to the results of studies, the prevalence of anxiety in the first, second, and third trimesters of pregnancy was 19.5%, 16.8%, and 17.2%, respectively [10]. Studies in Iran have reported a prevalence of pregnancy-related anxiety of 49.7% [11].

Women who are unable to access therapeutic abortion due to political and religious restrictions may experience significant anxiety and worry about the health and well-being of their fetus, as well as their ability to cope with the challenges [12]. Anxiety and worry during pregnancy can negatively affect the cognitive, emotional and behavioral development of children in addition to the physical and mental health of pregnant women [13]. Pregnant mothers experience some degree of stress before the test and while waiting to receive the results of the screening tests. Women who have intermediate and high-risk test results and need additional assessments will be more worried and anxious. Insufficient mother skills in dealing with these issues can be the main reason for the need to provide psychological services to cope with stress in this period of pregnancy [14].

Cognitive-behavioural counselling (CBC) is one of the psychological therapies that may lead to the improvement of anxiety disorders [15]. Despite the evidence supporting the effectiveness of CBC in the treatment of anxiety disorders in the general population, limited studies have been conducted on the specific effect of CBC on the treatment of anxiety during pregnancy [16]. Screening is one of the methods of care in pregnancy which cause anxiety and worry in pregnant women. In the meantime, the use of psychological interventions is important due to the lack of side effects and experimental support. Therefore, this study aimed to determine the effect of Cognitive Behavioral Counseling on the anxiety and worry levels of women with intermediate risk during first trimester screening for Down Syndrome.

## Methods

### Setting and design of the study

The study was a randomized controlled trial with two intervention and control groups. This study was performed on 52 pregnant women with intermediate risk (1: 51 − 1:1500) during first-trimester screening for Down Syndrome and without additional structural anomalies who were selected from urban and rural health centers of Abhar, Khorramdareh and Soltanieh cities located in Zanjan province, Iran, from September 2020 to August 2021.

### Participants

Based on the mean and standard deviation of worry scores in the intervention (24.18 ± 8.03) and control (17.55 ± 5.06) groups in the Kordi et al. study [17], power = %90, and error type 1 = 0.05, the sample size was calculated 21 people for each group. Considering the 20% attrition rate, 26 women are needed for each group.

During the initial antenatal visit, women are provided with verbal information regarding prenatal screening. Subsequently, after becoming acquainted with the details, informed consent is obtained from women regarding their decision to participate or decline the first trimester fetal abnormality screening test. The appropriate test for Iranian society should be performed in the first trimester so that there is a possibility of therapeutic abortion if there is an abnormality. Due to the prevailing religious and legal conditions in our country, medical abortion is permitted only up to 19th gestational weeks based on the LMP. Screening test for detecting 21, 13, and 18 trisomies in the first trimester of pregnancy includes Pregnancy Associated Plasma Protein A (A-PAPP), Beta - human chorionic gonadotrophin (FBHCG) and Fetal Nuchal Translucency (NT). Patients are divided into three categories: high risk (risk greater than 1:50), medium risk (between 1:51 − 1:1500) and low risk (risk less than 1:1501).

Inclusion criteria included pregnant women with intermediate risk (1: 51 − 1:1500) during first-trimester screening for Down Syndrome and without additional structural anomalies, willingness to participate in the study, literate, and gestational age less than 14 weeks. Exclusion criteria before allocation into two groups included unwillingness to continue the study, having a physical and mental illness or use of psychiatric medications, complications of pregnancy such as bleeding and suspected miscarriage, and molar pregnancy.

### Sampling methods

**Fig. 1:**
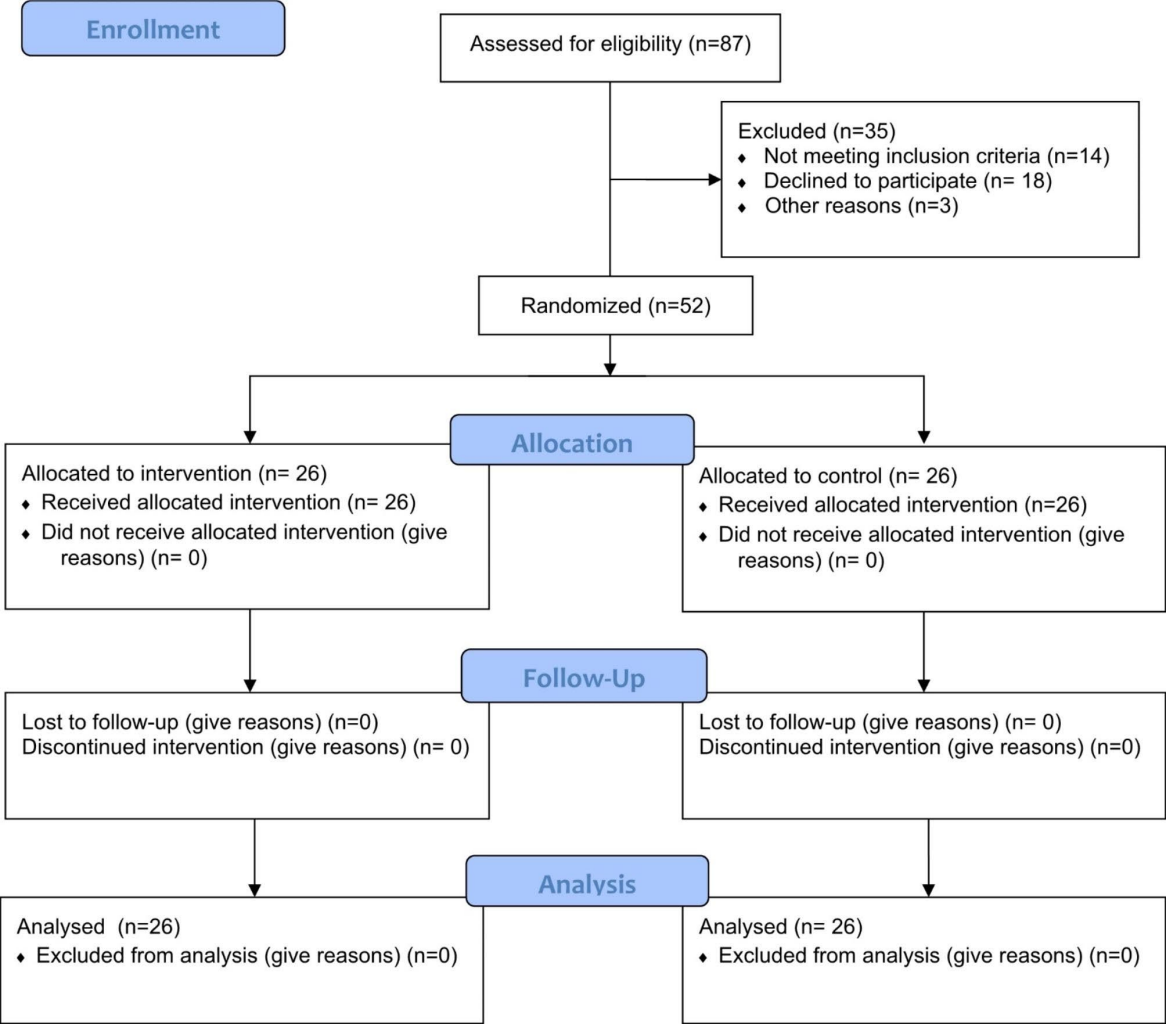
Consort Flow Diagram

Among 87 pregnant women evaluated by the researcher, 35 women have been excluded from the study for the following reasons: Not meeting inclusion criteria (n = 14), Declining to participate (n = 18), or Other reasons (n = 3). Finally, 52 women met the eligible criteria and were allocated to intervention (n = 26) and control (n = 26) groups using a four-block randomization method. First, all the four-block modes of the intervention (A) and control (B) groups were determined and the random allocation sequence was selected from a random numbers Table (13 blocks). The 52 envelopes were numbered and arranged in the same sequence and each letter of this sequence was placed in them. An envelope was opened to determine the assignment of an individual to either the control or intervention group, and the person was subsequently allocated to the designated group. The order and sequence of allocation were done by the research supervisor (RKh) and the participants were registered and enrolled by the research colleagues, and the participants were assigned to each group by the researcher (FV). See Fig. 1 for the process of sampling presented.

### Interventions

The intervention group received individual CBC in four sessions by phone in addition to routine care. Two sessions per week were held and each session lasted about 90–120 min. The counselling sessions were conducted by the second author who had passed a course on the Cognitive Behavioral Counseling method in a private clinic in Zanjan. Then, a Cognitive Behavioral Counseling program was developed by the researchers. The timing of the sessions was determined by the agreement of the participants. The control group received routine care that included nutritional counselling during pregnancy within the framework of the intervention group counselling. In our study, both groups were referred to a Perinatologist, for further treatment. According to the recommendation of the latest Iran Ministry of Health guideline, for the medium risk of the first-trimester screening tests the following two recommendations are acceptable. 1-Performing quad marker test in the 15th week of gestational age and combining it with the first trimester and reporting the unit risk in the form of a sequential protocol. 2- NIPT test.

It is mandatory to perform an anomaly scan in the 18th to 20th week of gestational age by measuring the markers in both recommendations even if the answer was normal.

Second-trimester screening tests put mothers in two risk groups. The first group are those who are in the high-risk group after measuring biochemical markers, and these women are also suggested to perform an invasive diagnostic test such as amniocentesis. The second group is low-risk people who do not need any other tests. To check the karyotype of the fetus, amniocentesis is performed in the second trimester. Termination of pregnancy is recommended if the karyotype result is abnormal.

All the study participants had the intermediate risk reported for Down syndrome and none of them had the risk for other anomalies. All mothers performed the second quad marker screening during the 15th to 17th week of pregnancy which showed that all of them were within the low-risk limit. One of the mothers used the NIPT screening with the opinion of a Perinatologist, which was negative for Down syndrome but it was positive for Klinefelter and the mother decided to keep the fetus. All of the participants were done anomaly scans in the 18th to 20th week of gestational age. There was no attrition in the study and after the interventions. The researchers and participants were not blinded.

### The first session

The first session included introducing and familiarity with treatment, identifying factors that motivate anxiety and worry, and completing the anxiety worksheet for the participant.

### The second session

The second session included reviewing the anxiety record sheet, teaching appropriate techniques to reduce anxiety and worry, identifying negative automatic thoughts, replacing positive thoughts and/or accepting the problem, and presenting homework.

### The third session

The third session included reviewing training of the previous sessions, introducing problem-solving skills and how to deal with avoided situations, distinguishing between the probability of occurrence and the possibility of occurrence, and presenting homework.

#### The fourth session

The fourth session included teaching self-efficacy skills, emphasizing on recognize and value positive traits, continuing self-reward, emphasizing the present, and gradual completing of treatment.

### Data collection tools

The primary outcome of the study was anxiety and the secondary outcome was a worry of pregnant women with prenatal screening tests suspected of fetal anomalies in the first trimester of pregnancy.

Data collection tools included a demographic and reproductive checklist, the Pregnancy-Related Anxiety Questionnaire (PRAQ), and Cambridge Worry Scale.

### Demographic and reproductive checklist

The demographic and reproductive checklist included the age of the mother and spouse, level of education, job, ethnicity and economic status, gestational age, number of pregnancies and deliveries, history of abortion, history of fetal anomaly, wanted or unwanted pregnancy, and type of deliveries.

### Pregnancy-related anxiety questionnaire (PRAQ)

The PRAQ measures pregnancy anxieties and was developed in 1989 by Vandenberg [18]. The short form of PRAQ has 17 items. The questionnaire includes five subscales including fear of childbirth (3 questions: 4,11,16), fear of giving birth to a child with physical or mental health issues (4 questions: 1,6,9,13), fear of changes in marital relationships (4 questions: 2, 8, 12, 14), fear of changes in mood and its consequences for the child (3 questions: 7, 10, 17), and fear of changes in the personal life of the mother (3 questions: 3,5,15). The total score of this questionnaire is obtained by adding the scores of all sub-scales. The score of each sub-scale is rated from one to seven based on the Likert scale. So, the total anxiety score was between 17 and 119. Huizink et al. (2004) [19]assessed the psychometric properties of the PRAQ and showed an acceptable correlation coefficient of it with the Spielberger state anxiety and trait questionnaire. Cronbach’s alpha of all sub-scales throughout pregnancy was reported to be above 0.76. Askarizadeh et al. showed a test-retest reliability coefficient on the scale of 0.65 to 0.72 in Iran [20].

### Cambridge worry scale

The Cambridge worry scale contained 16 questions with 6-point Likert response options ranging from no worries (score zero) to severe anxiety (score 5). The total score was obtained from the sum of the scores of the questions. The minimum score is zero and the maximum score is 80. This scale has four sub-scales including health (4 questions), socio-medical (5 questions), socio-economic (4 questions), and relationships (3 questions). Green (2003) confirmed its validity and reliability in the UK by the test-retest coefficient of 0.7 [21]. Yousefi showed the reliability of the Persian version of the Cambridge scale using Cronbach’s alpha coefficient of 0.78 in Iran [22].

### Data analysis and statistical tests

Data were described using descriptive statistical methods such as mean and standard deviation. Then, the normality of quantitative data was examined using the Kolmogorov-Smirnov test. All data had normal distribution except the sub-scales of the PRAQ and the Cambridge worry scale. To normalize the distribution of these variables, the rule expressed by Nazeri et al. (2014) was used [23]. Therefore, logx + 20 of the fear of changes in marital relationships, logx + 1 of the fear of changes in mood and its consequences for the child and logx + 25 of the relationships were calculated.

An Independent t-test was used to evaluate the differences between the intervention and control groups in terms of total anxiety and worry in different phases of the study. Intergroup changes of anxiety and worry and their sub-scales were analyzed using repeated measures of Analysis of Variance (ANOVA). SPSS version 16 was used for data analysis (P < 0.05).

## Results

The mean ± standard deviation (SD) of the age of participants in intervention and control groups was 34.34 (6.62) and 32.19(5.88), respectively. The education level of most participants in the intervention and control groups was high school and higher (73.07 and 69.22%) and the job of them was a housewife (84.61 and 80.76%). There was no statistically significant difference between the two groups in terms of demographic variables (Table 1).

**Table 1:** Demographic and reproductive variables of the participants in the intervention and control groups

Variables	Intervention		Control		P-Value
	Number	Percent	Number	Percent	
Age*	34.34	6.62	32.19	5.88	0.221
Spouse age*	36.84	6.12	37.00	6.48	0.919
Ethnicity (Turkish)	26	100	25	96.15	1.000
Place of Residence (City)	25	96.15	23	88.46	0.610
Education Elementary/Guidance school High school or university	7	26.92	8	30.76	0.689
	19	73.07	18	69.22	
Spouse education Elementary/Guidance school High school or university	10 16	38.46 61.54	9 17	34.61 65.39	0.942
Job (Housewife)	22	84.61	21	80.76	1.000
Spouse job Unemployed Clerk Worker Self-employed	1	3.84	1	3.84	0.927
	5	19.23	3	11.53	
	7	26.92	8	30.76	
	13	50.00	14	53.84	
Disease (No)	22	84.61	20	76.92	0.726
Taking medication (No)	21	80.76	21	80.76	1.000
Number of pregnancies One Two Three or more	5	19.23	6	23.07	0.854
	13	50.00	11	42.30	
	8	30.76	9	34.61	
Type of pregnancy (Wanted)	18	69.23	19	73.08	0.760
History of abortion (Yes)	6	23.07	9	34.61	0.358
History of fetal anomaly	0	0	0	0	-
Gestational age (LMP)*	94.15	4.83	94.73	3.74	0.956
Intermediate risk for Down Syndrome	26	100	26	100	1

*Mean (SD), LMP: Last Menstrual Period

In the counselling group, the mean (SD) of a total score of anxiety before the intervention was 67.11(20.68) which increased to 32.50 (13.58) six weeks after the intervention. Furthermore, the mean (SD) of a total score of worry before the intervention was 56.19 (16.76) which increased to 32.96(8.89) six weeks after the intervention.

At the baseline, there were no statistically significant differences in the mean of anxiety and worry between the intervention and control groups. There was a statistically significant difference between the two groups in terms of anxiety immediately after the intervention (P = 0.002) and at the 6-week follow-up (P = 0.008). Also, there was a statistically significant difference between the two groups in terms of worry immediately after the intervention (P = 0.001) and at the 6-week follow-up (P = 0.011) (Table 2).

**Table 2:** Comparison of anxiety and worry between the intervention and control groups during different phases of the study

Variables	Phase	Intervention		Control		P-Value
		Mean	SD	Mean	SD	
Anxiety	Before intervention	67.11	20.68	60.50	19.33	0.239
	After intervention	35.53	13.75	52.46	21.42	0.002
	6 weeks follow up	32.50	13.58	47.42	23.63	0.008
Worry	Before intervention	56.19	16.76	52.65	18.75	0.477
	After intervention	32.57	9.44	47.76	17.37	0.001
	6 weeks follow up	32.96	8.89	42.19	15.16	0.011

About anxiety and its subscales, repeated measures ANOVAs indicated that the fear of childbirth (F = 4.77 and P = 0.018), the fear of giving birth to a child with physical or mental health issues (F = 225.52 and P = 0.001), the fear of changes in marital relationships (F = 7.62 and P = 0.002), the self-centered fears or fear of changes in the personal life of the mother (F = 3.73 and P = 0.027), and the total anxiety score (F = 20.34 and P = 0.001) had a statistically significant decrease over time. The fear of changes in mood and its consequences for the child did not decrease significantly over time. Eta squared coefficient showed that 8.7% changes in the fear of childbirth, 33.8% changes in the fear of giving birth to a child with physical or mental health issues, 13.2% changes in the fear of changes in marital relationships, 6.9% changes in the self-centred fears or fear of changes in the personal life of the mother, and 28.9% of the total anxiety changes were due to the intervention (Table 3).

**Table 3:** Repeated measure analysis of variance of anxiety, worry, and subscales of them during different phases of the study

Variables	Within-subject effect	Sum of Squares	df	Mean Squares	F Time× Group	P-Value	Squared Eta	Test power
Fear of childbirth	Time × group	89.09	1.55	57.23	4.77	0.018	0.087	0.70
	Error	932.28	77.83	11.97				
Fear of giving birth to a child with physical or mental health issues	Time × group	571.47	2	285.73	225.52	0.001	0.338	1.00
	Error	1119.64	100	11.19				
Logarithm of fear of changes in marital relationships	Time × group	0.12	1.55	0.07	7.62	0.002	0.132	0.89
	Error	0.79	77.84	0.01				
Logarithm of fear of changes in mood and its consequences for the child	Time × group	0.95	2	0.47	2.48	0.089	0.047	0.48
	Error	19.19	100	0.19				
Self-centered fears or fear of changes in the personal life of the mother	Time × group	100.32	2	50.16	3.73	0.027	0.069	0.67
	Error	1344.00	100	13.44				
Total anxiety	Time × group	4428.51	1.73	2552.61	20.34	0.001	0.289	1.00
	Error	1088.41	86.74	125.50				
Health	Time × group	244.88	2	122.42	15.56	0.001	0.237	0.99
	Error	786.48	100	7.86				
Socio-medical	Time × group	310.97	1.19	260.90	8.77	0.003	0.149	0.87
	Error	1773.02	59.59	29.75				
Socio-economic	Time × group	99.24	1.65	60.02	7.76	0.002	0.134	0.91
	Error	638.71	82.67	7.72				
Logarithm of relationships	Time × group	0.03	1.70	0.01	4.05	0.028	0.075	0.65
	Error	0.39	85.27	0.00				
Total worry	Time × group	2380.88	1.49	1589.14	19.84	0.001	0.284	0.99
	Error	5997.46	74.91	80.06				

About worry and its subscales, repeated measures ANOVAs indicated that the health (F = 15.56 and P = 0.001), the socio-medical (F = 8.77 and P = 0.003), the socio-economic (F = 7.76 and P = 0.002), the relationships (F = 4.05 and P = 0.028), and the total worry score (F = 19.84 and P = 0.001) had a statistically significant decrease over time. Eta squared coefficient showed that 23.7% of changes in health, 14.9% changes of the socio-medical, 13.4% of changes in socio-economic, 7.5% of changes in relationships, and 28.4% changes in the total worry changes were due to intervention (Table 3).

## Discussion

The CBC was effective in the anxiety of pregnant women about prenatal screening tests. The intervention was also effective on the anxiety sub-scales including the fear of childbirth, the fear of giving birth to a child with physical or mental health issues, the fear of changes in marital relationships, and the fear of changes in the personal life of the mother. However, it was not effective on the subscale of fear of changes in mood and its consequences for the child. Moreover, the intervention was effective on the worry and all of its sub-scales. Pregnant women with anxiety identify potential health threats and exacerbate their anxiety and develop spontaneous, persistent fretfulness response patterns, thoughts, and feelings. The continuation of these types of thoughts, feelings and anxious behaviors eventually causes this chain to be completely out of consciousness [24–26].

This study revealed that individual CBC was effective on the total anxiety of pregnant women with intermediate screening results. This result is consistent with the results of many previous studies with different counselling approaches in Iran. Bayat et al. indicated that short-term psychological intervention is effective on anxiety in pregnant women with positive screening results [27]. Also, problem-oriented training is effective in the quality of life of pregnant women at risk of genetic abnormalities in the fetus [28]. Moreover, therapeutic coping reduces anxiety, depression, and physical symptoms and increases the social functioning and general health of pregnant women at high risk for fetal genetic abnormalities [14]. However, Khodakarami et al. showed that spiritual counselling is not effective in depression, anxiety, and stress in pregnant women. This difference can be attributed to the method and content of the counselling sessions. Khodakarami et al. did group counselling, while in the present study, the researchers did individual counselling. Despite the benefits that can be mentioned for group counselling, it seems that it has some limitations. For example, some participants are not willing to be in a group and some disrupt teamwork. Accordingly, group counselling may force group members to do something before they are ready for it, or it may require group members to disclose themselves. Also, in individual counselling, the participants have more opportunities to express their views and can easily state their questions and problems [29].

The results of this study showed that during different phases of the study, the sub-scales of anxiety except for the fear of changes in mood and its consequences for the child decreased significantly over time. Karrabi et al. (2019) also showed that solution-oriented counselling is effective on pregnant women’s concerns about fetal and maternal health and family relationships and childbirth [30]. HosseinKhanzadeh et al. (2017) also showed the effectiveness of the CBC on pregnancy anxiety, choice of type of delivery, and mental health of primiparous women [31]. The results of this study showed that the greatest effect of the intervention was on the fear of giving birth to a child with physical or mental health issues. Conversely, Vakilian et al. showed that acceptance and commitment therapy had no significant effect on women’s anxiety during pregnancy and fear of giving birth to a child with physical or mental health issues. One of the most common reasons for maternal fear of childbirth is due to fear of harm to the baby [32]. The reason for not being effective in an intervention based on acceptance and commitment can be attributed to cognitive integration. In such a way that thoughts are so intertwined with behaviours that it has kept the mother away from being in present and with her values [33]. It is suggested that more studies be done on the effect of this intervention on the fear of changes in mood and its consequences for the child in pregnant women.

The CBC was effective on the worry and its subscales. Consistent with this study, Kurdi et al. (2015) showed that group and individual training of pregnant women about screening tests is effective in anxiety and worry about pregnancy [17]. Cognitive-behavioural therapy focuses on techniques by which clients can retrieve and change their inner thoughts, especially thoughts related to emotional symptoms such as anxiety, depression, and anger. This treatment teaches clients to think about their thinking. The purpose of this technique is to change dysfunctional emotions and behaviors into functional ones. A recent systematic review and meta-analysis showed that irrespective of clinical status, age of participants, and delivery format, cognitive-behavioural and emotional interventions are effective for various problems [34]. So, it is suggested that the CBC be used in combination with prenatal screening to reduce the anxiety and worry of pregnant women during this period of pregnancy. Also, the inclusion of maternal support programs by midwifery consultants in health centers is recommended.

One of the limitations of this study is the self-reporting of anxiety and worry of pregnant women. It was out of the researcher’s control. Most of the participants were housewives, it is suggested Generalizability of the result was done with this limitation. Other limitations of the study were the specific circumstances of the country in terms of the coronavirus epidemic, which made it impossible to provide face-to-face counselling. Conducting similar studies with different approaches and longer follow-up time and duration are suggested.

## Conclusion

According to the results of the study, the CBC can reduce the anxiety and worry of pregnant women about screening tests for fetal anomalies. It seems that this method can reduce anxiety and its components by modifying cognitions.

### Electronic supplementary material

Below is the link to the electronic supplementary material.

Supplementary Material 1

## Data Availability

The dataset used in the present study is available from the corresponding author upon reasonable request.

## Abbreviations

CBC: Cognitive-Behavioural Counseling
SD: Standard Deviation
PRAQ: Pregnancy-Related Anxiety Questionnaire
ANOVA: Analysis of Variance
LMP: The last menstrual period
