# Supplementary material for: The effect of cognitive behavioral counseling on anxiety and worry level of women with intermediate risk during first trimester screening for down syndrome: a randomized controlled trial: a randomized controlled trial
Source: BMC Pregnancy Childbirth. 2023 Sep 1;23:630. doi: 10.1186/s12884-023-05857-2 (PMC10472680; doi:10.1186/s12884-023-05857-2)
Supplement: Supplementary file 1 — Supplementary Material 1 [file 12884_2023_5857_MOESM1_ESM.docx]

**The short form of the Pregnancy-Related Anxiety Questionnaire (PRAQ)**

(Backward translated from Persian to English form)

Please read each sentence carefully and circle the number that best suits your situation according to the instructions below.

1. It is not true at all
2. It is hardly acceptable.
3. It is more wrong than right.
4. On average, it is true.
5. It is partially true.
6. It is acceptable to a considerable extent.
7. It is absolutely true.

| **Row** | **The item** | **1** | **2** | **3** | **4** | **5** | **6** | **7** |
| --- | --- | --- | --- | --- | --- | --- | --- | --- |
|  | I am afraid that my child will not be healthy. | 1 | 2 | 3 | 4 | 5 | 6 | 7 |
|  | I am a little worried that our baby might be less attractivel and I am afraid of the reaction of others. | 1 | 2 | 3 | 4 | 5 | 6 | 7 |
|  | I am concerned about my sudden mood changes. | 1 | 2 | 3 | 4 | 5 | 6 | 7 |
|  | I fear the pain during labor and delivery. | 1 | 2 | 3 | 4 | 5 | 6 | 7 |
|  | I am concerned about my irritability. | 1 | 2 | 3 | 4 | 5 | 6 | 7 |
|  | I am concerned about my unattractive physical appearance. | 1 | 2 | 3 | 4 | 5 | 6 | 7 |
|  | I am worried about unpleasant changes in my child's appearance. | 1 | 2 | 3 | 4 | 5 | 6 | 7 |
|  | I am worried that I will have to miss many things because of the baby. | 1 | 2 | 3 | 4 | 5 | 6 | 7 |
|  | I am afraid that my child has brain damage or mental retardation. | 1 | 2 | 3 | 4 | 5 | 6 | 7 |
|  | I am worried that my body will not return to its normal shape after giving birth. | 1 | 2 | 3 | 4 | 5 | 6 | 7 |
|  | I am afraid of unknown things in pregnancy and childbirth. | 1 | 2 | 3 | 4 | 5 | 6 | 7 |
|  | I worry that becoming a mother will change me too much and make me feel old. | 1 | 2 | 3 | 4 | 5 | 6 | 7 |
|  | I am worried that my child will die before birth. | 1 | 2 | 3 | 4 | 5 | 6 | 7 |
|  | I am worried that my child will become troublesome and make excuses. | 1 | 2 | 3 | 4 | 5 | 6 | 7 |
|  | I am concerned about becoming preoccupied with myself and I'm worried that I might become isolated. | 1 | 2 | 3 | 4 | 5 | 6 | 7 |
|  | I'm worried that I might scream and lose control during labor. | 1 | 2 | 3 | 4 | 5 | 6 | 7 |
|  | I am worried about getting fat. | 1 | 2 | 3 | 4 | 5 | 6 | 7 |

**Cambridge Worry Scale** (Backward translated from Persian to English form)

Often we worry about something. We'd love to know if any of the following has worried you at all. For each statement, please circle the item that shows how much it worries you right now. If anything else worries you or if you would like to say something more about any of the above, please write here. .....

| **Very much** | **Much** | **Medium** | **Low** | **Very little** | **Never** | **The item** | **Row** |
| --- | --- | --- | --- | --- | --- | --- | --- |
| Very much | Much | Medium | Low | Very little | Never | The health of the fetus |  |
| Very much | Much | Medium | Low | Very little | Never | Giving birth |  |
| Very much | Much | Medium | Low | Very little | Never | The possibility of miscarriage |  |
| Very much | Much | Medium | Low | Very little | Never | Employment problems |  |
| Very much | Much | Medium | Low | Very little | Never | Your housing |  |
| Very much | Much | Medium | Low | Very little | Never | Going to the hospital |  |
| Very much | Much | Medium | Low | Very little | Never | The health of someone close to you |  |
| Very much | Much | Medium | Low | Very little | Never | Financial problems |  |
| Very much | Much | Medium | Low | Very little | Never | Internal examinations |  |
| Very much | Much | Medium | Low | Very little | Never | Coping with the new baby |  |
| Very much | Much | Medium | Low | Very little | Never | Your own health |  |
| Very much | Much | Medium | Low | Very little | Never | Giving up work |  |
| Very much | Much | Medium | Low | Very little | Never | Whether your spouse will be with you for the birth |  |
| Very much | Much | Medium | Low | Very little | Never | Communication with spouse |  |
| Very much | Much | Medium | Low | Very little | Never | Problems with the law |  |
| Very much | Much | Medium | Low | Very little | Never | Relationship with your family and friends |  |
